# Functional characterization of the active *Mutator-*like transposable element, *Muta1* from the mosquito *Aedes aegypti*

**DOI:** 10.1186/s13100-016-0084-6

**Published:** 2017-01-11

**Authors:** Kun Liu, Susan R. Wessler

**Affiliations:** 1 Graduate Program in Botany and Plant Sciences, University of California, Riverside, CA 92521 USA; 2 Department of Botany and Plant Sciences, University of California, Riverside, CA 92521 USA

**Keywords:** Transposable elements, *Mutator*-like elements (MULE), *Aedes aegypti*, Yeast assay, Target site duplication (TSD), Transposase

## Abstract

**Background:**

*Mutator*-like transposable elements (MULEs) are widespread with members in fungi, plants, and animals. Most of the research on the MULE superfamily has focused on plant MULEs where they were discovered and where some are extremely active and have significant impact on genome structure. The maize *MuDR* element has been widely used as a tool for both forward and reverse genetic studies because of its high transposition rate and preference for targeting genic regions. However, despite being widespread, only a few active MULEs have been identified, and only one, the rice *Os3378*, has demonstrated activity in a non-host organism.

**Results:**

Here we report the identification of potentially active MULEs in the mosquito *Aedes aegypti.* We demonstrate that one of these, *Muta1*, is capable of excision and reinsertion in a yeast transposition assay. Element reinsertion generated either 8 bp or 9 bp target site duplications (TSDs) with no apparent sequence preference. Mutagenesis analysis of donor site TSDs in the yeast assay indicates that their presence is important for precise excision and enhanced transposition. Site directed mutagenesis of the putative DDE catalytic motif and other conserved residues in the transposase protein abolished transposition activity.

**Conclusions:**

Collectively, our data indicates that the *Muta1* transposase of *Ae. aegypti* can efficiently catalyze both excision and reinsertion reactions in yeast. Mutagenesis analysis reveals that several conserved amino acids, including the DDE triad, play important roles in transposase function. In addition, donor site TSD also impacts the transposition of *Muta1*.

**Electronic supplementary material:**

The online version of this article (doi:10.1186/s13100-016-0084-6) contains supplementary material, which is available to authorized users.

## Background

Transposable elements (TEs) are mobile fragments of DNA that can move from one locus to another in the host genome, often replicating in the process. TEs usually make up the largest fraction of eukaryotic genomes; accounting for almost half of the human genome, and more than 70% of the genomes of some grass species [1]. Based on their transposition intermediate, eukaryotic TEs are divided into two classes. Class 1 elements utilize an RNA intermediate in the transposition reaction while the intermediate for most class 2 elements is the element itself that is mobilized by a 'cut and paste' mechanism [2]. A TE family is composed of one or more transposase-encoding autonomous elements and up to several thousand nonautonomous elements that do not encode functional transposase. Family members share the same terminal inverted repeats (TIRs) and target site duplications (TSDs) of the same length allowing them to move in *trans* by utilizing the transposase encoded by the autonomous family member(s) [3].

Prior studies have classified class 2 TEs into upwards of 19 superfamilies on the basis of the relatedness of the element-encoded transposase [4]. The original *Mutator* element, now called *MuDR*, was first isolated from a maize strain as the agent responsible for its high forward mutation rate [5, 6]. Subsequently, members of this superfamily, called collectively *Mutator*-like transposable elements (MULEs), were found in other plants, and in fungi, protozoans, and in a variety of animals, (from insects to fish to other metazoans) [3, 7–9]. Typical features of MULEs include long terminal inverted repeats (TIRs) (>100 bp) and an 8-10 bp TSD [10]. Nonautonomous family members often contain a variety of sequences between the TIRs, including fragments from host genes; such elements are called Pack-MULEs [11]. To date, only a few active MULEs have been identified, including *MuDR*, *Hop1*, Jittery, *TED*, and *Os3378* [6, 12–15]. Importantly, only the rice *Os3378* element has been shown to transpose in a heterologous host [15].

Most MULE transposases contain a N-terminal zinc finger DNA binding motif and a conserved C-terminal DDE domain, which has been shown to be the catalytic core for transposition reactions in other superfamilies but not to date for MULEs [16, 17]. Phylogenetic studies indicate that the DDE domain of MULE transposases is closely related to the IS*256* family, which is present in diverse prokaryotes [18]. Each residue of the predicted DDE motif of IS*256* has been shown to be necessary for transposition [19]. Most MULE transposases also harbor a CH motif and a W residue between the second D and E of the DDE domain, which are also conserved in the *hAT* superfamily. Mutagenesis analyses in *hAT* elements demonstrated the importance of these residues for transposition [20, 21]. Whether these residues are also important for the MULE transposase has not as yet been determined.

TE-based genetic tools have facilitated our deep understanding of the biology of both plants and animals, however, such tools are not currently available in the mosquito species, including *Aedes aegypti, Aedes albopictus, Culex quinquefasciatus* and *Anopheles gambiae,* which can spread dengue fever, yellow fever, chikungunya, zika, and many other diseases that are responsible for over one million deaths annually [22–25]. Four exogenous TEs that transpose at high frequencies in *Drosophila melanogaster* (*Hermes, Mos1, Minos*, *piggyBac)* were found to move rarely or not at all in the germ line of mosquitoes as very few germinal mutations were detected [26, 27]. To explain this result, it was hypothesized that mosquitoes have a strong genome defense system that could effectively recognize and silence foreign TEs [28]. Therefore endogenous active TEs could be effective mutagens from generation to generation as they might be able to evade genome surveillance. Availability of the genome sequence enabled the search for potentially active TEs in the mosquito genomes [29–32]. In this study we show that *Muta1,* identified by computer-assisted analysis of the *Ae. aegypti* genome, is an active TE capable of both insertion and excision in a yeast transposition assay. Site-directed mutagenesis analysis revealed that transposition activity in yeast was influenced by disruption of several conserved residues and by the presence of TSDs at the donor site. With characteristics such as high transposition activity, precise excision, and no target sequence preference, *Muta1* could be crafted into an effective tool for forward mutation analyses in mosquitoes.

## Results

### Distribution of the MULE superfamily in mosquito genomes

To identify potentially active MULEs, we first assessed the abundance and distribution of MULEs in the genomes of *Ae. aegtpti, Ae albopictus, C quinquefasciatus*, and *An. gambiae* [29–32]. The conserved MULE DDE domain sequences were used as queries in TBlastN searches against the *Ae. aegypti* genome through the TARGeT program, which is designed for TE discovery [20, 33]. After removal of duplicate hits, sequences with significant similarity (e- value <10^-15^) to the MULE DDE domain were identified and used to build a phylogenetic tree. As a result, no significant hits were identified in the *An. gambiae* genome, whereas 141, 105, and 10 putative MULE DDE domain sequences were identified from the *Ae. aegtpti, Ae albopictus,* and *C quinquefasciatus* genomes, respectively (Additional file 1: Figure S1).

**Fig. 1:**
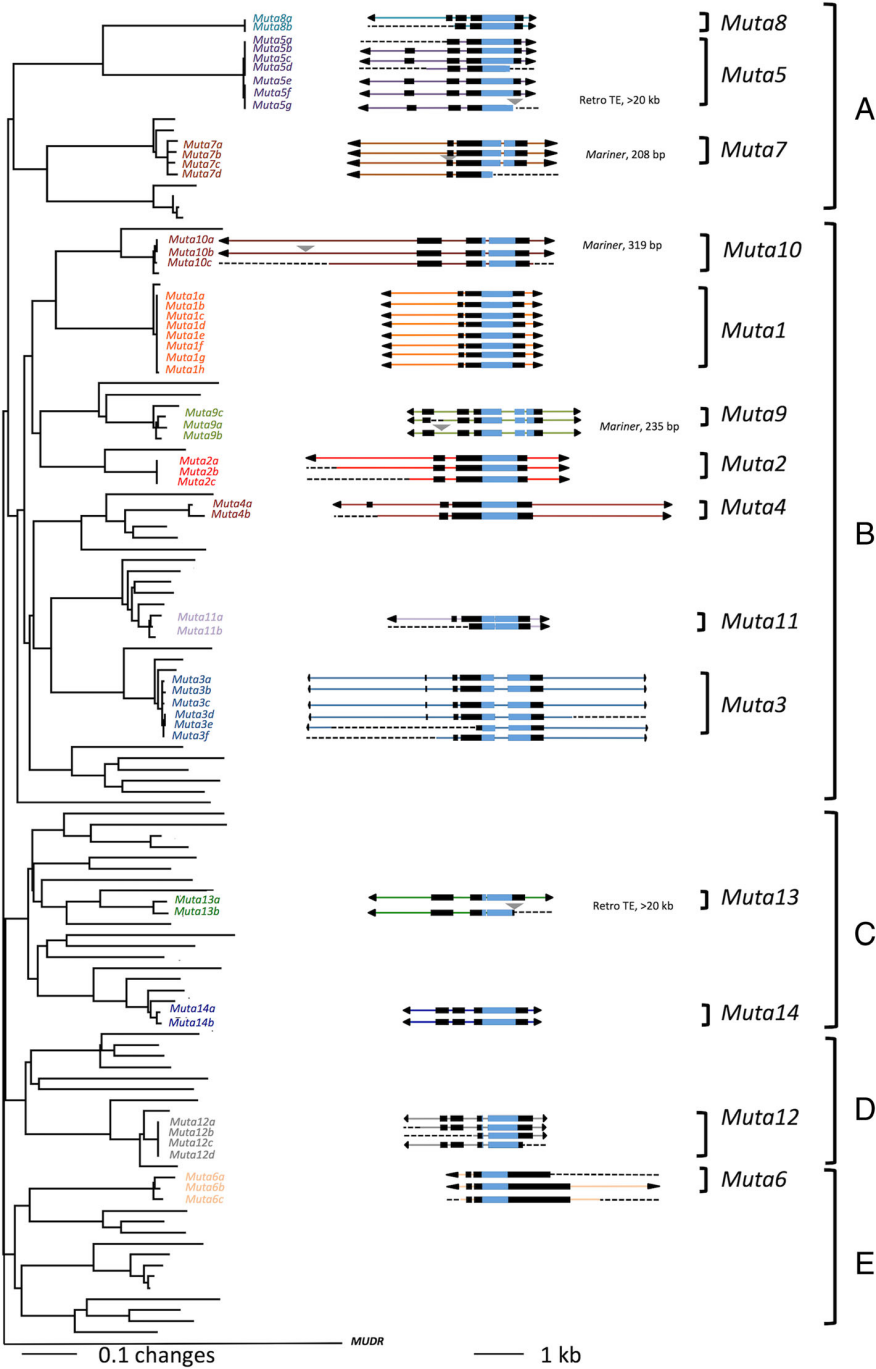
Phylogenetic tree and structure of MULEs in *Ae. aegypti.* Neighbor-joining tree generated from a multiple alignment of 141 conceptually translated catalytic domains from transposase proteins with bootstrap values calculated from 1000 replicates. For element structures: TIRs are black triangles, exons are black boxes, DDE domains are blue boxes, introns are lines connecting boxes, colored lines indicate within family identity of noncoding regions, other TE insertions are gray triangles above elements, and dashed lines are missing sequences caused by gaps, deletions or large insertions. The maize *MuDR* transposase is used as an outgroup. The *Ae. aegypti Mutator* transposases are classified into 5 major lineages **a**–**e**

Full-length elements were defined by comparison of sequences in the same branch of the phylogenetic tree where adjacent sequences share high similarity that extends beyond the DDE domain. Sequences near the boundary of similarity were examined manually for the long TIRs and 8-10 bp TSDs that are features of MULEs. The 10 hits in *C. quinquefasciatus* were likely remnants of ancient insertion events as they are highly divergent in sequence and lack identifiable TIR and TSD. The quality of the *Ae. albopictus* genome assembly prevented our search for full-length elements. In the *Ae. aegypti* genome, 31 full-length elements sorted into 14 families, each with > 95% sequence similarity (*Muta1-14,* Fig. 1).

### Characterization of the MULE superfamily in *Ae. aegypti*

All of the 14 MULE families identified from *Ae. aegypti* have 8-9 bp TSD, 12 of 14 have TIRs >100 bp and two have TIRs <60 bp. Ten of the 12 long TIR families contain short subterminal tandem repeats (9-15 bp). All families except *Muta6* also contain derivative nonautonomous elements (Additional file 2: Table S1), which share high sequence similarity in their TIR and subterminal regions, but carry heterogeneous internal sequences including fragments of host genes.

The phylogenetic tree also reveals the evolutionary relationships of the 14 families. Among the 5 major groups resolved on the tree, group B includes half of the 14 families, while group D and E contains one family each (Fig. 1). Furthermore, long branches indicate extensive sequence differences between families. For example, although *Muta5* and *Muta8* belong to group A and locate to adjacent clades, no significant nucleotide similarity was detected between these elements. In another example, nucleotide similarity between *Muta3* and *Muta11* is restricted to the DDE region (71% identity, ~390 bp) and the TIRs (76% identity, ~ 110 bp).

After removal of other TE insertions and manual correction of frameshifts caused by small insertions and deletions, the 31 full-length elements were predicted to encode transposase proteins ranging in size from 416 to 554 residues (Additional file 2: Table S1). Comparison of conceptually translated transposases identified conserved regions, other than the DDE domain, that could have functional significance. The transposases of 13 families were predicted to harbor a FLYWCH type zinc finger DNA binding domain in the N-terminus, while *Muta6* was predicted to harbor a SWIM type zinc finger DNA binding domain in the C-terminus. Each family contains at least one putative full-length copy in which the coding region is not interrupted by frameshifts or stop codons. Of particular interest to this study, 3 families (*Muta1, Muta3* and *Muta5*) include at least 2 members that are identical or nearly identical (>99% identity). These features indicate recent and possible ongoing activity of multiple MULE families in *Ae. aegypti*.

### Identification of the active *Muta1* family

**Fig. 2:**
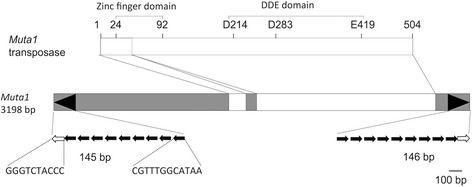
Structural features of the *Muta1* element and its transposase. The eight virtually identical *Muta1* elements contain noncoding regions (shaded) and the coding region (white boxes) for the predicted 504-residue transposase with the predicted zinc and catalytic (DDE) domains discussed in the text. Structural features of *Muta1* include its distinctive long TIR (black arrowheads) whose substructure, expanded at the bottom, includes the 10 bp terminal palindromic motif (open arrow) and the 12 bp subterminal tandem repeats (black arrows) with linker DNA of 3-4bp represented by gaps between solid arrows

Of the 14 families, *Muta1* appeared to be the best candidate for an active element. The family contains 7 identical copies and an 8th copy with only two point mutations in predicted noncoding DNA. *Muta1* is 3198 bp, flanked by TSDs of 8 bp or 9 bp, TIRs of 145 bp comprised of a 10 bp imperfect palindromic terminal motif with the 5th and 6th nucleotide unpaired, and 9 copies of a 12 bp subterminal tandem repeat separated by 3-4 bp spacers (Fig. 2, open and solid arrows, respectively). Because of the complexity of the TIR structure, *Muta1* can be classified as a type2 *Foldback* TE [34]. The *Muta1* transposase is predicted to be 504-amino acid transposase and encoded by two exons.

**Fig. 3:**
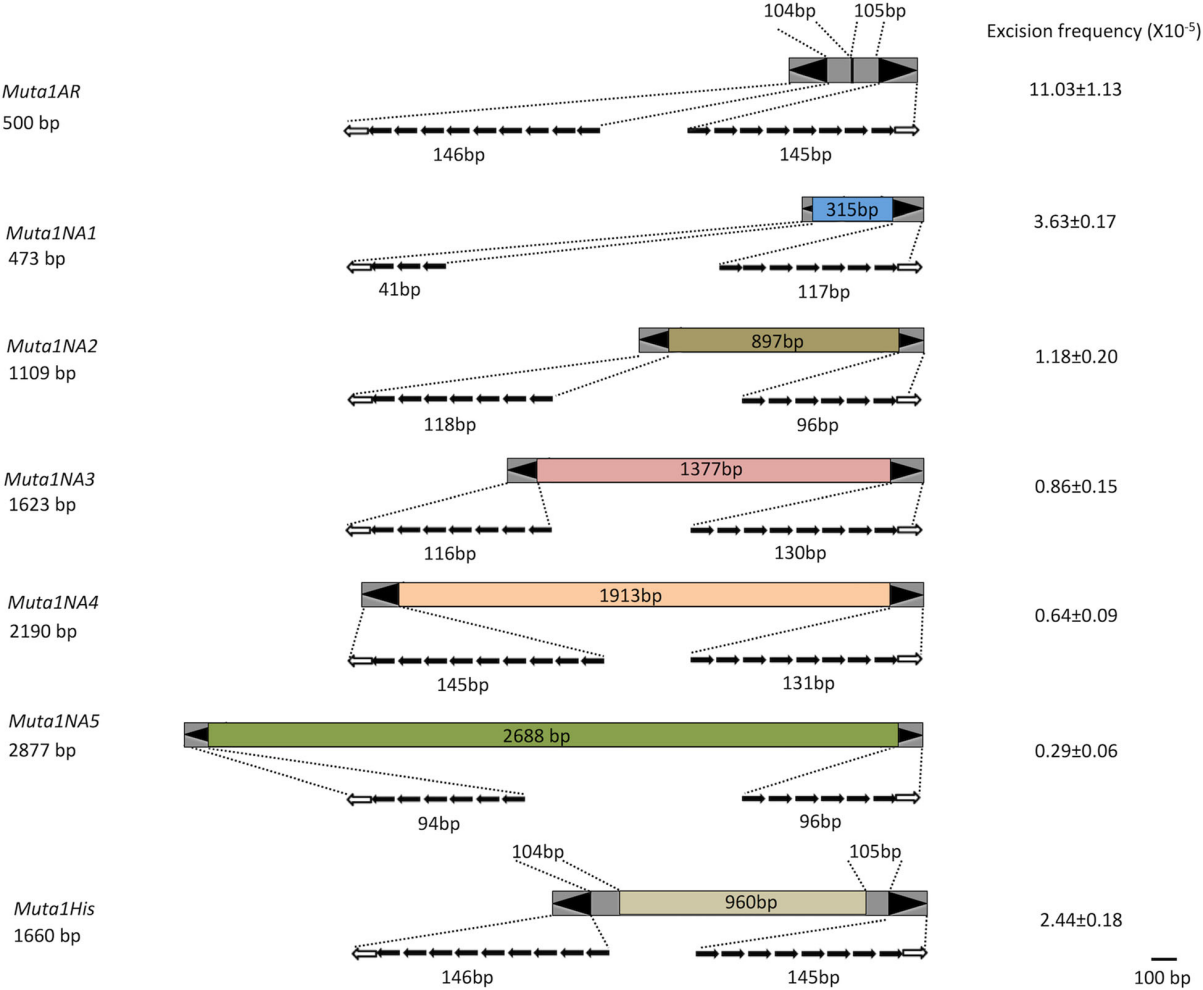
Structural features of nonautonomous *Muta1* elements used in this study. *Muta1NA1* through *Muta1NA5* are natural elements; *Muta1AR* and *Muta1His* are artificial. Element lengths and internal sequences are indicated. Black arrowheads represent the TIRs, which include the terminal palindromic motif (open arrow) and subterminal tandem repeats (solid arrows). Gray shaded regions are sequences derived from *Muta1*; colored regions of each element indicate the diverse origin of internal sequences. Excision frequencies from the *ADE2* reporter in yeast assays are on the right

Over 300 putative nonautonomous elements derived from *Muta1* were detected in the *Ae. aegypti* genome (e- value <10^-10^), with 171 flanked by perfect TSDs of 8 bp or 9 bp (Additional file 3: Figure S2A). Most of the derivative elements share the TIR sequence with *Muta1,* but the TIRs are often truncated, with variable copies of the subterminal repeats (Fig. 3). There are multiple copies of some derivative elements; for example, there are 4 copies of *Muta1NA1* (>98% identical). Most derivative elements contain variable internal sequences and share sequence similarity with only the TIR of *Muta1*. For about 20% of the derivative elements these variable internal regions can be aligned with sequences from host genes, much like previously described Pack-MULEs [11]. For example, the 1623 bp *Muta1NA3* contains a 276 bp fragment from a serine/threonine-protein kinase gene (97.5% identical, e-value < 1e-51) (Fig. 3). Among the 171 insertion sites, 110 (64.3%) are located in gene bodies or within 5kb upstream or downstream of genes. In a control dataset of 171 randomly selected genomic sites (see methods) only 49 (28.7%) were located in the same regions (Additional file 3: Figure S2B), suggesting that *Muta1* may have an insertion preference for genic regions as was previously reported for plant MULEs [6].

### *Muta1* can transpose in yeast

A yeast transposition assay was employed to determine whether *Muta1* transposase is able to catalyze the movement of natural and/or artificial nonautonomous elements. In prior studies members of five superfamilies (MULE, *Tc1/mariner, hAT, PIF/Harbinger* and *piggyBac*) were shown to transpose in yeast [15, 35–38]. Our yeast assay consisted of two plasmid constructs: an expression vector containing the *Muta1* transposase coding sequence downstream of the galactose inducible *GAL1* promoter, and a reporter vector containing a nonautonomous element inserted in, and blocking expression of, the *ADE2* gene (Additional file 4: Figure S3A). We first tested the artificial *Muta1AR* element which contains 250 bp from each end of *Muta1* (Fig. 3). Transposase mediated TE excision restored *ADE2* expression and resulted in reversion of adenine auxotrophy to prototrophy (Additional file 4: Figure S3B, Additional file 5: Figure S4A). Excision events were validated by PCR amplification of the *ADE2* empty site from revertants (Additional file 6: Figure S5A).

In subsequent experiments, the reporter plasmid was modified by inserting several natural nonautonomous elements (*Muta1NA1-5*, Fig. 3, Additional file 5: Figure S4B-F) into the *ADE2* coding region. Despite differences in TIR length and the length and sequence of internal regions, *Muta1* transposase was able to mobilize all of these elements, but none with a frequency as high as the artificial element *Muta1AR*.

Integration events catalyzed by *Muta1* transposase were assayed by first constructing the nonautonomous *Muta1HIS* element containing a yeast selectable marker *HIS3* gene flanked by 250 bp of the *Muta1* termini (Fig. 3). Integration of *Muta1HIS* into yeast chromosomes was assayed in plasmid-free cells following selection with 5-FOA, (see Methods, Additional file 4: Figure S3D). Comparison of the frequency of *ADE2* revertants retaining the *HIS3* marker and are 5-FOA resistant (2.71 × 10^−6^ His^+^ 5-FOA^R^ cells/total cells) to the frequency of total *ADE2* revertants (2.44 × 10^−5^ Ade^+^ cells/total cells) indicated that about 11% of the *Muta1HIS* elements excised from the donor plasmid had reintegrated in yeast chromosomes (Table 1). In another assay, *ADE2* colonies isolated directly from the *Muta1HIS* excision assay were tested to determine if they were also His^+^ and 5-FOA^R^. Of 300 revertant colonies, 41 (14%) could proliferate on selective plates, in agreement with the results of the first approach (Table 1).

**Table 1:** *Muta1* integration in yeast

Assay	Frequency
*Muta1His* integration^a^	
ade2^+^ excisant	2.44 × 10^−5^ Ade^+^ cells/total cells
His^+^ 5-FOA^R^ excisant	2.71 × 10^−6^ His^+^ 5FOA^R^ cells/total cells
	ratio of reintegration = 11%
*Muta1His* integration^b^	
ade2^+^ excisant	300 colonies
His^+^ 5-FOA^R^ excisant	41 colonies
	ratio of reintegration = 14%

^a^Measured by selection for presence of *Muta1His* and absence of plasmid following excision from *ADE2*

^b^Measured by analysis of independent Ade^+^ revertants to maintain *Muta1His* but lacking plasmid

To determine the precise insertion sites, polymorphic bands on transposon display gels were recovered [39], sequenced and mapped to yeast chromosomes (Additional file 6: Figure S5B). Sixty of 62 sites had significant matches with yeast genomic sequence, while two matched plasmid sequences. Fourteen of the 60 genomic sites were in gene bodies while 27 were within 1kb of genes. Thus 41/60 insertions (68%) were in gene-rich regions [40]. Amplification and sequencing of each site revealed the presence of TSDs with 8 bp for 21 events and 9 bp for 39 events (Additional file 7: Table S2). Consensus sequences generated from the yeast insertion sites and the 171 sites for *Muta1* derivative elements in the *Ae. aegypti* genome indicated little or no sequence preference for insertion (Additional file 8: Figure S6).

### The *Muta5* element does not transpose in yeast

The successful transposition of *Muta1* in yeast prompted us to perform a similar analysis of the *Muta5* family which contains 3 identical copies, a 4th with >99% sequence identity, and 3 copies with large deletions or insertions (Fig. 1). *Muta5* is 3496 bp, flanked by TIRs of 151 bp and TSDs of 8 bp or 9 bp and is predicted to contain 3 exons that encode a 554-amino acid transposase. The TIR of *Muta5* contains 9 copies of a 15 bp subterminal tandem repeat and an 8bp terminal motif (Additional file 9: Figure S7A). There are over 200 *Muta5* derivative elements (e- value <10^-15^) in the *Ae. aegypti* genome, with 93 flanked by 8 bp or 9 bp TSD. Taken together the features of the *Muta5* family strongly suggested that it was an active element. However, in the yeast transposition assay the *Muta5* transposase was unable to catalyze transposition of *Muta5AR* (Additional file 9: Figure S7A)*,* which contains 250 bp from the ends of *Muta5* (Additional file 9: Figure S7B)*.*

### TSD at donor site affects *Muta1* transposition in yeast

Successful transposition of *Muta1* in yeast facilitated the analysis of the importance of its features by quantifying the impact of mutations on transposition quality and frequency. With regard to the role of the TSDs, although the nonautonomous constructs used in the assays described thus far lacked flanking TSDs, their reinsertion still generated TSDs of 8 bp or 9 bp. To examine the impact of TSD length or sequence on excision frequency of *Muta1AR*, three versions of 8 bp (TTCAATAG, CGATTCAA and GGTAACTC) or 9 bp (ATTCAATAG, TCGATTCAA and CGGTAACTC) TSDs were tested. Addition of 8 bp or 9 bp TSDs at the donor site increased *Muta1AR* excision frequency by ~7 fold and ~3 fold, respectively when compared to the controls lacking TSDs (Table 2). Similarly, introduction of TSDs flanking *Muta1HIS* increased reintegration by about 40% and 90% for 8 bp TSDs or 9 bp TSDs respectively (Table 2). For both excision and integration, TSD sequence had little impact.

**Table 2:** Impact of TSD on transposition

TSD length	TSD sequence	Excision	Reintegration	precise excision
		frequency (X 10 ^-5^)	frequency (%)^a^	/examined excision
0bp		11.03 ± 1.13	13.9 ± 1.8	4/38
	TTCAATAG	63.34 ± 9.12	20.88 ± 1.53	25/29
8bp	CGATTCAA	76.33 ± 11.53	18.22 ± 2.90	27/31
	GGTAACTC	68.53 ± 12.16	19.15 ± 2.39	24/27
	ATTCAATAG	31.52 ± 5.27	27.83 ± 3.01	27/29
9bp	TCGATTCAA	36.43 ± 6.12	24.35 ± 1.98	28/30
	CGGTAACTC	28.38 ± 3.11	29.01 ± 2.76	26/30

^a^Measured by analysis of independent Ade^+^ revertants to maintain *Muta1His* but lacking plasmid

We next addressed the question of whether the presence or absence of TSDs at the donor site impacts the quality of excision by analyzing so-called transposon footprints. Specifically, class 2 TEs often leaves a footprint upon excision consisting of a few nucleotides or small rearrangements at the site of excision site [41]. Formation of footprints involves DNA repair of sequences flanking the excised element. To assess the impact of TSDs on the repair of excision sites, the donor element construct was modified so that all excision footprints (not only those that maintain the reading frame) could be analyzed. First, nonautonomous elements were inserted in the 5’ UTR of *ADE2* (Additional file 4: Figure S3C). Second, because insertion of *Muta1AR* in the 5’UTR resulted in leaky *ADE2* expression, we substituted the longer *Muta1NA1* (Fig. 3), which, in the absence of transposase, blocked *ADE2* expression. When either 8 bp or 9 bp TSDs were added to *Muta1NA1*, about 90% of revertants were precise (Table 2), meaning that the element was removed as well as a single copy of the TSD. The quality of excision appeared to be independent of TSD sequence (Additional file 10: Figure S8B-G). In contrast, absence of donor site TSDs reduced perfect excision to only 10% of the *ADE2* revertant colonies sequenced (Table 2). Most excision sites reflected loss of a few nucleotides from either side of the flanking DNA. Occasionally, part of the TIR (up to 13 bp) was left after excision and repair (Additional file 10: Figure. S8A).

### Mutagenesis of *Muta1* transposase

**Fig. 4:**
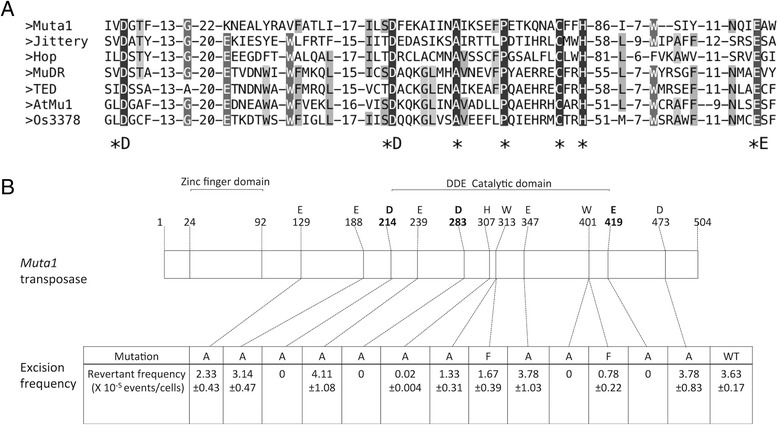
MUSCLE alignment of the DDE domain in MULE transposases and the impact of mutations. **a** MUSCLE alignment of the DDE domain in *Muta1, MuDR, TED, atMu1, Os3378, Jittery,* and *Hop*. Shaded residues have related physical or chemical properties with darker shading denoting more conservation. Asterisks denote residues conserved in all sequences. **b** Schematic of the 504 amino acid Muta1 transposase with positions of the putative FLYWCH type zinc finger DNA binding domain (24–92 aa), and DDE triad (residues D214, D283, E419). Designated amino acids were mutated to alanine (*A*) or to both alanine and phenylalanine (*F*) resulting in the excision frequencies shown below. See text for details

The catalytic domains of all characterized transposases of class 2 TEs contain a DDE/D amino acid triad [20] and mutagenesis studies confirmed its functional significance in the *piggyBac, Mariner* and *hAT* superfamilies [38, 42, 43]. Alignment with the transposase of other active MULE*s* showed that the DDE triad in *Muta1* corresponds to D214, D283 and E419 (Fig. 4a). To determine if these conserved sites play key roles in transposition, site-directed mutagenesis was performed. Transposition activity was completely abolished when D214, D283 or E419 was mutated to alanine (Fig. 4b, Additional file 5: Figure S3I-K). In contrast, mutation of nonconserved sites, including E129, E188, E239, W313 and D473 to alanine had little impact. Although E373 is not a conserved site, mutation to alanine also completely abolished transposition activity.

The functional significance of two additional highly conserved residues in *Muta1*, H307 and W401, were tested. In a prior study these residues were shown to be essential for transposition of the *hAT* superfamily member *Hermes* [21, 43]. Mutation of the corresponding *Hermes* H and W residues (H268 and W319) to alanine completely abolished transposition activity. Similarly for the *Muta1* transposase*,* mutation of H307 or W401 to alanine abolished activity (Fig. 4b). Analysis of *Hermes* transposase also showed that the W319 residue was likely necessary for the correct positioning of flanking DNA during the excision reaction, and that other aromatic residues can partially substitute for this function [21]. When W401 of *Muta1* was mutated to phenylalanine, transposition activity was reduced by 79% (Fig. 4b, Additional file 5: Figure S3L) and the frequency of precise excision dropped from 90% to approximately 48% (8 bp donor site TSD, 14/29 events) and 50% (9 bp donor site TSD, 15/30 events) (Additional file 10: Figure S8H&I), suggesting that the *Muta1* W401F mutation also led to inaccurate positioning. These results confirmed the importance of the putative DDE motif, the conserved H307, W401 and identified the nonconserved E373 as a potentially important residue for transposase function.

## Discussion

The MULE superfamily is widespread in eukaryotic genomes and is closely related to prokaryotic IS*256* elements. However, although it is also found in the genomes of many insects, no active elements have been reported in insect species. In this study, we performed a thorough search for potentially active MULEs in the *Ae. aegypti* genome and demonstrate that *Muta1* encodes a transposase that catalyzes the excision and reinsertion of nonautonomous derivative elements in yeast. With the availability of this heterologous transposition assay, the function of the conserved MULE DDE domain and the role of TSD in transposition were tested.

The DDE/D domain is proposed to be the catalytic core involved in transposition of class 2 TEs and has been identified in all superfamilies [20]. Prior to this study, the functional significance of this domain had been experimentally validated only for members of the *piggyBac*, *Tc1/Mariner*, and *hAT* superfamilies [38, 42, 43]. Results of this study provide the first experimental evidence for the importance of the DDE motif in the transposition reaction of *Muta1*, a member of the MULE superfamily. Specifically, transposition was completely abolished when any of the three residues were mutated to alanine (Fig. 4b, Additional file 5: Figure S3I-K).

In addition to the DDE triad, other residues critical for transposition were identified. W401 of *Muta1* is a conserved residue that is also found in the *hAT* superfamily [20, 43]. Crystallographic analysis and in vitro biochemical assays showed that the corresponding W318 residue in *Hermes* functions in the positioning of flanking DNA, which ensures that the double strand break occurs at the correct position when an element excises from flanking DNA [21]. Other aromatic residues partially substitute for its function, however the mutant transposase generated additional species of intermediates in double strand break repair than the wild-type transposase, suggesting that inaccuracy in the position of the cleavage site may be the cause by these mutations [21]. For *Muta1,* the W401A mutation completely abolished transposition activity while the W401F mutation resulted in a 79% reduction of transposition frequency (Fig. 4b, Additional file 5: Figure S3L) and caused inaccurate excision as the frequency of precise excision dropped from 90% to ~50% (Additional file 10: Figure S8H&I). Taken together these data suggest that this conserved tryptophan residue is likely playing a similar role in *hAT* and MULE transposases, which is to correctly position flanking DNA for the excision reaction. In addition to the W residue, a CxxH motif is also shared between MULE and *hAT* elements, the *Hermes* H268 was found to be located close to the DDE active center and involved in the interaction with TIRs [21]. Mutation of the corresponding H307 to alanine resulted in a 99.5% reduction of *Muta1* transposition activity (Fig. 4b), suggesting the importance of this residue for *Muta1* transposase function. Taken together, our study provides experimental evidence to support the close evolutional relationship reported previously between the MULE and *hAT* superfamilies [20].

Prior to this study, the only MULE shown to transpose in a heterologous host was the rice *Os3378* element [15]. Because both *Muta1* and *Os3378* have demonstrated activity in yeast assays that employed very similar experimental design and nonautonomous elements of similar size, comparison of assay results may be informative (Table 3). Excision frequencies of the 500 bp *Muta1AR*, as high as 6940 events per 10^7^ cells (Fig. 2), is ~320 fold higher than *Os3378NA469* (469bp). For both elements, excision frequencies are increased by the presence of donor site TSD with *Muta1AR* enhanced by ~7 fold (8 bp) and 3 fold (9 bp), and *Os3378NA469* enhanced by ~17 fold with TSDs of 9 bp (Table 3). About 80% of *Os3378* integration sites in the yeast genome were located in gene bodies or within 1 kb of flanking regions of genes while *Muta1* had a slightly lower ratio of 68%. In summary *Muta1* shows very similar transposition behavior as *Os3378,* and the higher activity of *Muta1* makes it a better tool for the future study of MULE transposition, for example, the biochemical process of excision and integration, and how Pack-MULEs capture host gene fragments [11].

**Table 3:** Comparison of *Muta1* and *Os3378* transposition in yeast

	*Muta1*	*OS3378*
Excision frequency no TSD (X 10^-7^)^a^	1103	1.2
Excision frequency 8bp TSD (X 10^-7^)^a^	6940	-
Excision frequency 9bp TSD (X 10^-7^)^a^	3211	20.2
Reintegration frequency No TSD^b^	13.90%	59.26%
Reintegration frequency 8bp TSD^b^	19.41%	-
Reintegration frequency 9bp TSD^b^	27.06%	39.28%
Percentage of reinsertion in gene rich regions	80%	68%

^a^Based on the excision of *Muta1AR* or *Os3378NA469* from the coding region of *ADE2*

^b^Based on the reintegration of *Muta1His* or *Os3378NA469*

- Corresponding experiment was not reported

The presence of donor site TSDs impacts the quality of *Muta1*-mediated excision events as various footprints were generated without donor TSD (Additional file 10: Figure S8A). The predominance of small deletions (1-4 bp) suggests that the *Muta1* transposase cuts outside the TIR. In contrast, with the presence of either 8 bp or 9 bp TSD at the donor site, most excision events were precise and the actual TSD sequence did not seem to matter (Additional file 10: Figure S8 B-G). Similar behavior was also observed for IS*256*, the prokaryotic TE family related to MULEs, and for the one other MULE tested, Os3378. Reduction of TSD from 8bp to 6bp eliminated precise excision of IS*256* and reduced the *Os3378* precise excision frequency from 97.44% to 82.05% [15, 19]. For IS*256* it was hypothesized that precise excision is achieved through a transposase-independent replication slippage mechanism that requires a short stretch of homologous DNA with a minimum length of 8 bp [44]. In our assay, the absence of donor TSDs resulted in a 90% reduction in precise excision (Additional file 10: Figure S8A-G), which suggests a role for TSDs in promoting precise excision.

Thirty-one full-length MULEs that group into 14 families were identified in the *Ae. aegypti* genome. Several families have identical or nearly identical full-length copies including *Muta1* (7 identical), *Muta5* (3 identical), and *Muta3* (2 with only 2 noncoding SNPs). Although the existence of identical genomic copies is a feature of active TEs, *Muta5* was unable to catalyze the movement of nonautonomous derivative elements in yeast (Additional file 9: Figure S7). One explanation for our success with *Muta1* but failure with *Muta5* is that the latter has 3 predicted exons while the former has 2. More predicted exons would increase the chances of incorrectly assembling the actual/functional *Muta5* transposase.

Accumulation of seven identical copies of *Muta1* in *Ae. aegypti* suggests that this element may still be active or that it has some success evading the genome surveillance system shown previously to effectively silence exogenous TEs [26–28]. In this regard, it may be possible to engineer *Muta1* to make it an effective endogenous mutagen. Like the *MuDR* system in maize, where the genome has numerous copies of nonautonomous *Mu* elements, there are over 300 *Muta1* derivative elements in the *Ae. aegypti* genome (Additional file 3: Figure S2A)*.* Although mobility in the yeast assay does not guarantee mobility in the host, if *Muta1* was able to mobilize even a subset of these elements as it does in yeast, it could be an effective tool for high frequency insertional mutagenesis, especially when coupled with its preference for genic insertions and a lack of target sequence preference (Additional file 3: Figure S2B, Additional file 8: Figure S6).

## Conclusions

This is the first report of the transposition of a non-plant MULE, *Muta1,* in a heterologous system and provides the first experimental evidence for the functional significance of the DDE domain in the transposition reaction in the MULE superfamily. High frequency transposition in a yeast assay facilitated the determination of *Muta1* transposition features including precise excision, genic targeting with no sequence preference and the impact of TIR and TSD for insertion and excision. Taken together, *Muta1* may be a valuable tool for forward genetics in mosquitoes.

## Methods

### Identification of MULEs in *Ae. aegypti*

The mosquito genomes used in this study: AaegL3 build for *Ae. aegypti* (https://www.vectorbase.org/organisms/aedes-aegypti/liverpool/aaegl3); AaloF1 build for *Ae. albopictus* (https://www.vectorbase.org/organisms/aedes-albopictus/foshan/aalof1); CpipJ2 build for *C. quinquefasciatus* (https://www.vectorbase.org/organisms/culex-quinquefasciatus/johannesburg/cpipj2) and AgamP4 build for *An. gambiae* (https://www.vectorbase.org/organisms/anopheles-gambiae/pest/agamp4). The conserved MULE DDE domain from all eukaryotes [20] was used as query to search the mosquito genomes by TBLASTN, as implemented in the TARGeT pipeline [33] with an E-value cutoff of 0.001. Flanking DNA sequences with 10 kb upstream and downstream of the matched region were retrieved. The ends of a putative element were determined by aligning two closely related elements with their 20 kb flanking sequences, TIR boundaries and TSDs were manually identified. Coding capacity of each element was predicted by the GENSCAN program (http://genes.mit.edu/GENSCANinfo.html).

To identify *Muta1* derivative nonautonomous elements, 50 bp from each end of *Muta1* was used in a BLASTN search with TARGeT [33] using default parameters. One hundred bp of flanking DNA sequences were retrieved for manual verification of the TIR and TSD of each derivative element. Fifty bp flanking each element were used for BLASTN searches against the *Ae. aegypti* genome (AaegL3 build) to determine the genomic location and compared to the genome annotation (release AaegL3.3, https://www.vectorbase.org/organisms/aedes-aegypti/liverpool/aaegl3) to determine the adjacent genes. For the control data set, 171 genome coordinates across the 4,757 scaffolds were randomly generated, and compared to the genome annotation (release AaegL3.3) to determine the surrounding sequences and genes. The random insertion sites generation used 1,000 replicates to estimate the expected number of insertions (and standard deviations) in each category.

### Yeast construct construction

Genomic DNA of individual *Ae. aegypti* mosquito (Liverpool strain, obtained from Dr. Atkinson, UC Riverside) was extracted using the DNeasy Blood & Tissue Kit (Qiagen). The two exons of *Muta1* predicted by GENSCAN program were cloned from genomic DNA and fused through overlap PCR (all primer sequences are available in Additional file 11: Table S3). The complete transposase coding sequence was then cloned into the Gateway cloning vector pENTR and transferred to destination vector pGAL415-ccdb [45] with LR Clonase (Invitrogen) to generate the pGAL415-ccdbMuta1 plasmid (Additional file 3: Figure S2A).

Two hundred fifty bp from each end of *Muta1* were fused by overlap PCR to generate *Muta1AR.* The *HIS3* fragment containing the yeast *HIS3* coding sequence, *HIS3* 5’ and 3’ UTR, and *HIS3* promoter was cloned from vector pGAL415-ccdb [45]. The *HIS3* fragment was then fused with 250 bp from each end of *Muta1* through overlap PCR to generate *Muta1HIS. Muta1NA1-5* elements were cloned directly from genomic DNA. All nonautonomous elements were inserted in the *Hpa*I site of *ADE2* for the exon excision assay or the *Xho*I site for 5’ UTR excision assay through homologous recombination in yeast as previously described [37]. Donor site TSDs were introduced by adding corresponding TSD sequences in primers (Additional file 11: Table S3).

For *Muta5* assay, plasmid pGAL415-ccdbMuta5 was constructed in the same way as pGAL415-ccdbMuta1, and *Muta5NA* was constructed by overlap PCR (Additional file 11: Table S3).

### Transposition assay

The yeast transposition assay using *Saccharomyces cerevisiae* strain DG2523 and the pWL89a vector was described previously [35, 36]. Transformation was performed using the Frozen-EZ Yeast Transformation kit (Zymo research). For excision assays, transformants were grown in 5 ml liquid media of CSM -leu-ura with 2% dextrose. After growth to saturation (36 h), cells were washed twice with 5 ml water, resuspended in 0.5 ml water and plated onto CSM -his-leu-ade with 2% galactose. Colonies were counted after incubation at 30°C for 15 days and viable counts were made by plating 100 μl of a 1 × 10^5^ and 1 × 10^6^ dilution on YPD plates.

For the reintegration assay, cells were grown to saturation in 5ml liquid CSM -leu-ura with 2% dextrose, cells were washed twice with 5 ml sterile water, resuspended in 0.5 ml water and plated onto CSM -leu-ura-ade with 2% galactose plate and CSM -his-leu + 5-FOA with 2% galactose plates. Colonies were counted after incubation at 30°C for 15 days, and viable counts were made by plating 100 μl of a 1 × 10^5^ and 1 × 10^6^ dilution on YPD plates. In another approach, individual Ade^+^ *Muta1HIS* excision revertant colonies isolated directly from plates of CSM -his-leu-ade with 2% galactose were streaked on CSM -his + 5-FOA plates to calculate the reintegration frequency.

### Excision and reinsertion analysis

For footprint analysis, colony PCR was performed on *ADE2* revertant colonies using primers (Table S3) flanking the insertion sites. PCR products were gel extracted (Zymoclean Gel DNA Recovery Kit) and sequenced. For reinsertion analysis, transposon display was conducted [39]. Genomic DNA was extracted from revertant colonies using the Yeastar genomic DNA kit (Zymo research); DNA samples were digested by *Bfa*I followed by adapter ligation. Pre-amplification and selective amplification were used to amplify the sequences between *Muta1* TIR and the *Bfa*I adapter sequence. Amplicons consisting of flanking sequences of the reinsertion sites and part of *Muta1* TIR were resolved on a 4% agarose gel, and polymorphic fragments were recovered and sequenced. Flanking sequences were mapped to the yeast genome (S288C, http://yeastgenome.org/) and the reinsertion sites were determined with regard to the closest genomic features. The insertion site analysis figure was made using the program Pictogram http://genes.mit.edu/pictogram.html.

### Mutagenesis of *Muta1* transposase

Site-directed mutagenesis was used to generate mutant versions of *Muta1* transposase. One pair of primers (Additional file 11: Table S3) was used for each mutation site, and pGAL415-ccdbMuta1 plasmid was used as template. PCR products were digested with *Dpn1* to remove template, and the resulting plasmid was sequenced to confirm that mutations occurred as expected.

## Additional files

Additional file 1: Figure S1. Phylogeny and MULE copy number of mosquito and fruit fly species. Phylogenetic relationship and approximate divergence time of *Ae.aegypti, Ae. albopictus, C. quinquefasciatu,s* and *An. gambiae.* Genome size and copy number of MULE DDE domain in each species is shown. (TIFF 292 kb)
Additional file 2: Table S1. Summary of 14 MULE families in *Ae. aegypti*. (DOCX 81 kb)
Additional file 3: Figure S2. Features of *Muta1* derivative elements in *Ae. aegypti*. (A) 171 *Muta1* derivative elements divide into 5 groups based on size. Within each group, the number of elements with 8 bp TSD and 9 bp TSD are shown in grey and black, respectively. (B) Distribution of *Muta1* derivative element insertion sites in the *Ae. aegypti* genome. Mean ± s.d., n = 1,000 (for control). Number of insertion sites in the *Ae. aegypti* genome and control data set is shown in black and grey, respectively. (TIF 4425 kb)
Additional file 4: Figure S3. Yeast transposition assay constructs. (A) Structures of pMuta1_PAG415 and pWL89Ae. AmpR, ampicillin resistance gene; ori, *E. coli* replication origin; Pgal1, GAL1 promoter; CYC1 ter, terminator; CEN, centromere sequences of yeast chromosomes; ARS, autonomous replication site. Dashed lines indicate the position of nonautonomous element insertions, in the 5’UTR and coding region respectively. Black arrows indicate the positions of primers used for PCR analysis in Figure S3A. (B) Excision from coding region of *ADE2*. (C) Excision from 5’ UTR of *ADE2*. (D) Reintegration. In the parental strain, pWL89A carries *Muta1HIS* in the coding region of *ADE2*. Reintegration is assayed by selecting cells that retain the *HIS* marker in Muta1HIS when the parental plasmid is excluded by 5-FOA treatment, which is toxic to Ura^+^ cells. (TIF 602 kb)
Additional file 5: Figure S4.
*ADE2* revertant colonies from the yeast transposition assay. (A-G) Excision activity of nonautonomous elements from the *ADE2* coding region. (H) Negative control, *Muta1AR* excision from the *ADE2* coding region is tested on plates without galactose. (I-L) *Muta1AR* excision from the *ADE2* coding region is tested with mutant transposases. (TIF 3512 kb)
Additional file 6: Figure S5. Analysis of excision and reinsertion events. (A) PCR analysis of the *Muta1NA1* excision sites from ADE2 revertants using flanking primers. Expected band size is 820bp (control) or 350bp, with or without *Muta1NA1,* respectively. (B) Transposon display analysis of *Muta1HIS* reinsertion in the yeast genome. DNA bands are amplicons consisting of flanking sequences of the reinsertion sites and part of the TIR. PWL89A-Muta1HIS vector is used as control. Arrows indicate the polymorphic bands that represent the insertion of *Muta1HIS* in different genomic locations. (TIF 413 kb)
Additional file 7: Table S2. Target Site Duplications (TSDs) and locations of *Muta1* transpositions in yeast. (DOCX 96 kb)
Additional file 8: Figure S6. Seqlogo of insertion sites of *Ae. aegypti Muta1* derivative elements and reintegration sites in yeast. Both 8bp TSDs (A) and 9bp TSDs (B) and their 7bp flanking sequences are analyzed, insertion preference is shown as a pictogram (height of letter indicates percentage of each nucleotide at that position) and the frequencies of preferred nucleotides, if any, are shown. (TIF 1361 kb)
Additional file 9: Figure S7 Structural features of *Muta5* and excision assay results. (A) Structural features of *Muta5*. White boxes are coding regions, shaded boxes are noncoding regions, and triangles are the TIR. Within the TIR, black arrows represent 9 copies of the 15bp subterminal tandem repeat, open arrow represents the 8bp terminal motif. The putative 554-residue transposase is predicted to harbor a zinc finger domain and the catalytic (DDE) domain. The artificial *Muta5AR* element contains 250 bp from each end of *Muta5*. (B) Excision frequencies of *Muta5AR* and *Muta5AR* from the *ADE2* reporter in the yeast assay. *Muta1* serves as the positive control. (TIF 373 kb)
Additional file 10: Figure S8. Footprints from *Muta1NA1* excision events. Arrows indicate the *Muta1NA1* insertion, length and sequence of the TSD in each assay (shown above the arrows). TSD or sequences derived from TSDs are in bold, sequences derived from *Muta1NA1* are underlined, the number of recovered events is on the right. (A) Footprints of *Muta1NA1* excision from the *ADE2* 5’ UTR without donor site TSD. (B-D) Footprints of *Muta1NA1* excision from the *ADE2* 5’ UTR with different 8 bp TSD sequences. (B) TTCAATAG; (C) CGATTCAA; (D) GGTAACTC. (E-G) Footprints of *Muta1NA1* excision from the *ADE2* 5’ UTR with different 9 bp TSD sequences. (E) TCGATTCAA, (F) CGGTAACTC, (G) ATTCAATAG. (H-I) Footprints of *Muta1NA1* excision from the *ADE2* 5’UTR with the transposase W401F mutation. (H) the 8bp TSD TTCAATAG was used. (I) the 9bp TSD ATTGAATAG was used. (TIF 665 kb)
Additional file 11: Table S3. Primers used in this study. (DOCX 124 kb)

## Abbreviations

MULE: Mutator-like transposable elements
PCR: Polymerase chain reaction
TE: Transposable element
TIR: Terminal inverted repeat
TSD: Target site duplication
