# Supplementary figures and images for: Functional characterization of the active Mutator-like transposable element, Muta1 from the mosquito Aedes aegypti
Source: Mob DNA. 2017 Jan 11;8:1. doi: 10.1186/s13100-016-0084-6 (PMC5225508; doi:10.1186/s13100-016-0084-6)

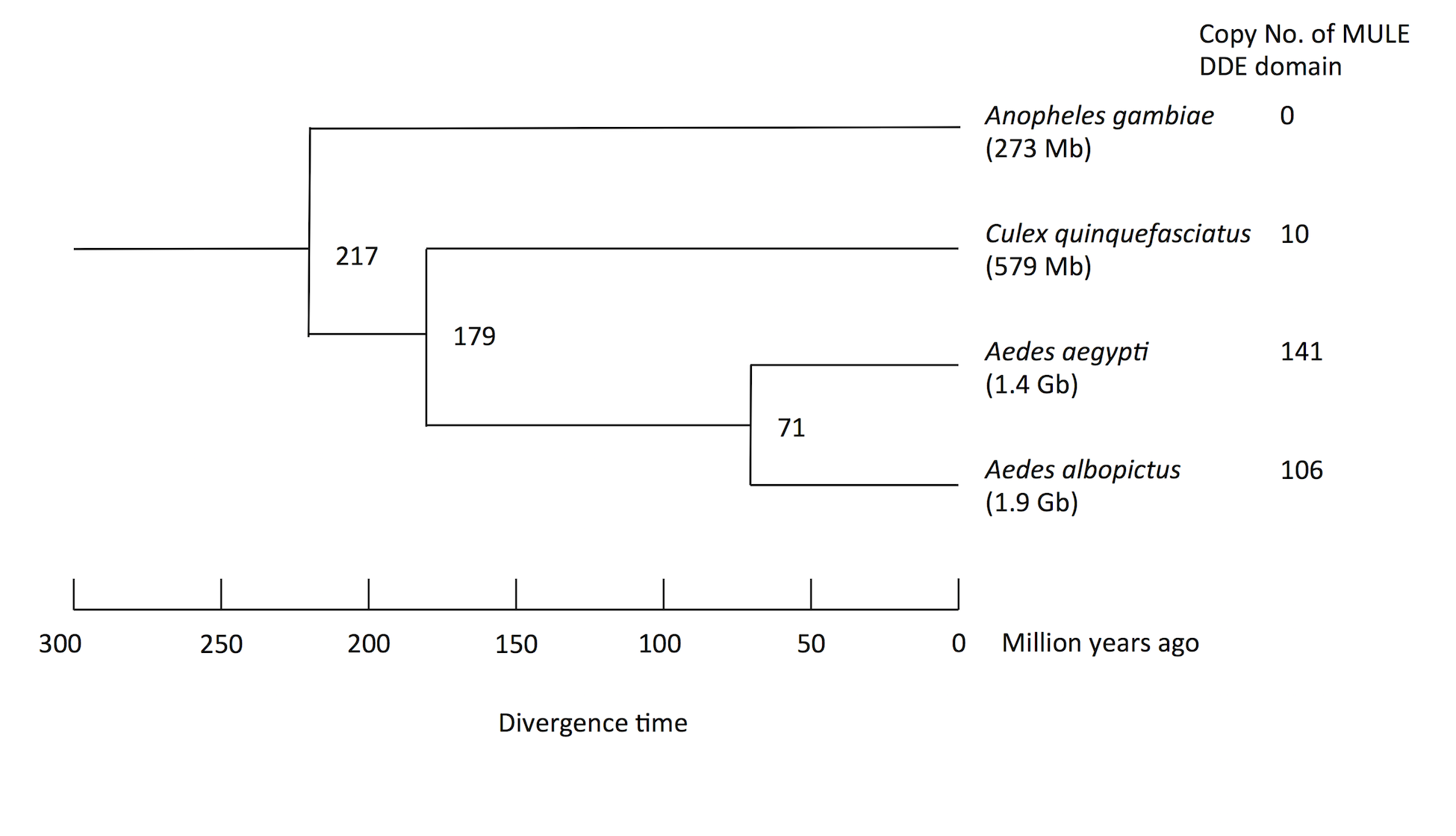

Supplement: Additional file 1: Figure S1. — Phylogeny and MULE copy number of mosquito and fruit fly species. Phylogenetic relationship and approximate divergence time of Ae.aegypti, Ae. albopictus, C. quinquefasciatu,s and An. gambiae. Genome size and copy number of MULE DDE domain in each species is shown. (TIFF 292 kb) [file 13100_2016_84_MOESM1_ESM.tiff]

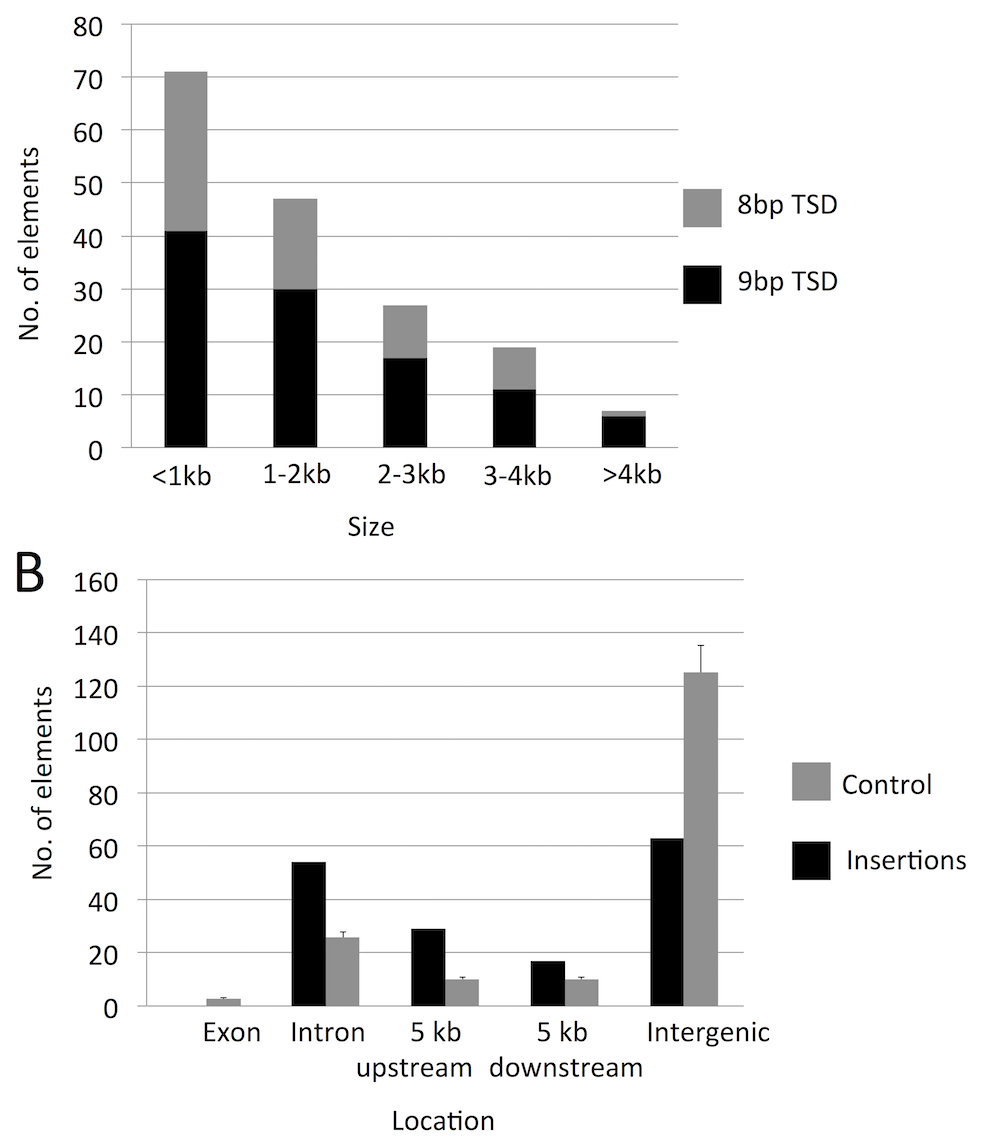

Supplement: Additional file 3: Figure S2. — Features of Muta1 derivative elements in Ae. aegypti. (A) 171 Muta1 derivative elements divide into 5 groups based on size. Within each group, the number of elements with 8 bp TSD and 9 bp TSD are shown in grey and black, respectively. (B) Distribution of Muta1 derivative element insertion sites in the Ae. aegypti genome. Mean ± s.d., n = 1,000 (for control). Number of insertion sites in the Ae. aegypti genome and control data set is shown in black and grey, respectively. (TIF 4425 kb) [file 13100_2016_84_MOESM3_ESM.tif]

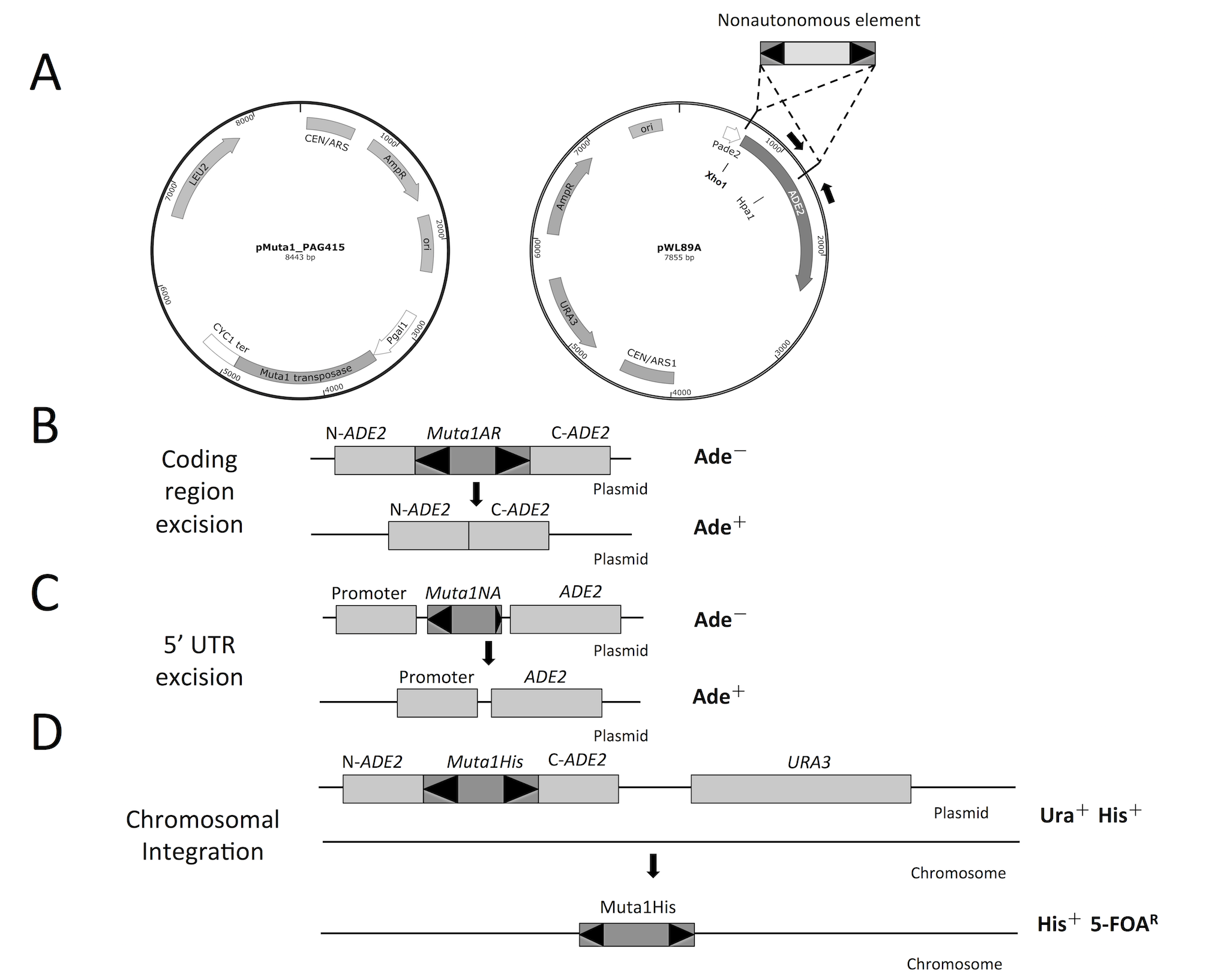

Supplement: Additional file 4: Figure S3. — Yeast transposition assay constructs. (A) Structures of pMuta1_PAG415 and pWL89Ae. AmpR, ampicillin resistance gene; ori, E. coli replication origin; Pgal1, GAL1 promoter; CYC1 ter, terminator; CEN, centromere sequences of yeast chromosomes; ARS, autonomous replication site. Dashed lines indicate the position of nonautonomous element insertions, in the 5’UTR and coding region respectively. Black arrows indicate the positions of primers used for PCR analysis in Figure S3A. (B) Excision from coding region of ADE2. (C) Excision from 5’ UTR of ADE2. (D) Reintegration. In the parental strain, pWL89A carries Muta1HIS in the coding region of ADE2. Reintegration is assayed by selecting cells that retain the HIS marker in Muta1HIS when the parental plasmid is excluded by 5-FOA treatment, which is toxic to Ura+ cells. (TIF 602 kb) [file 13100_2016_84_MOESM4_ESM.tif]

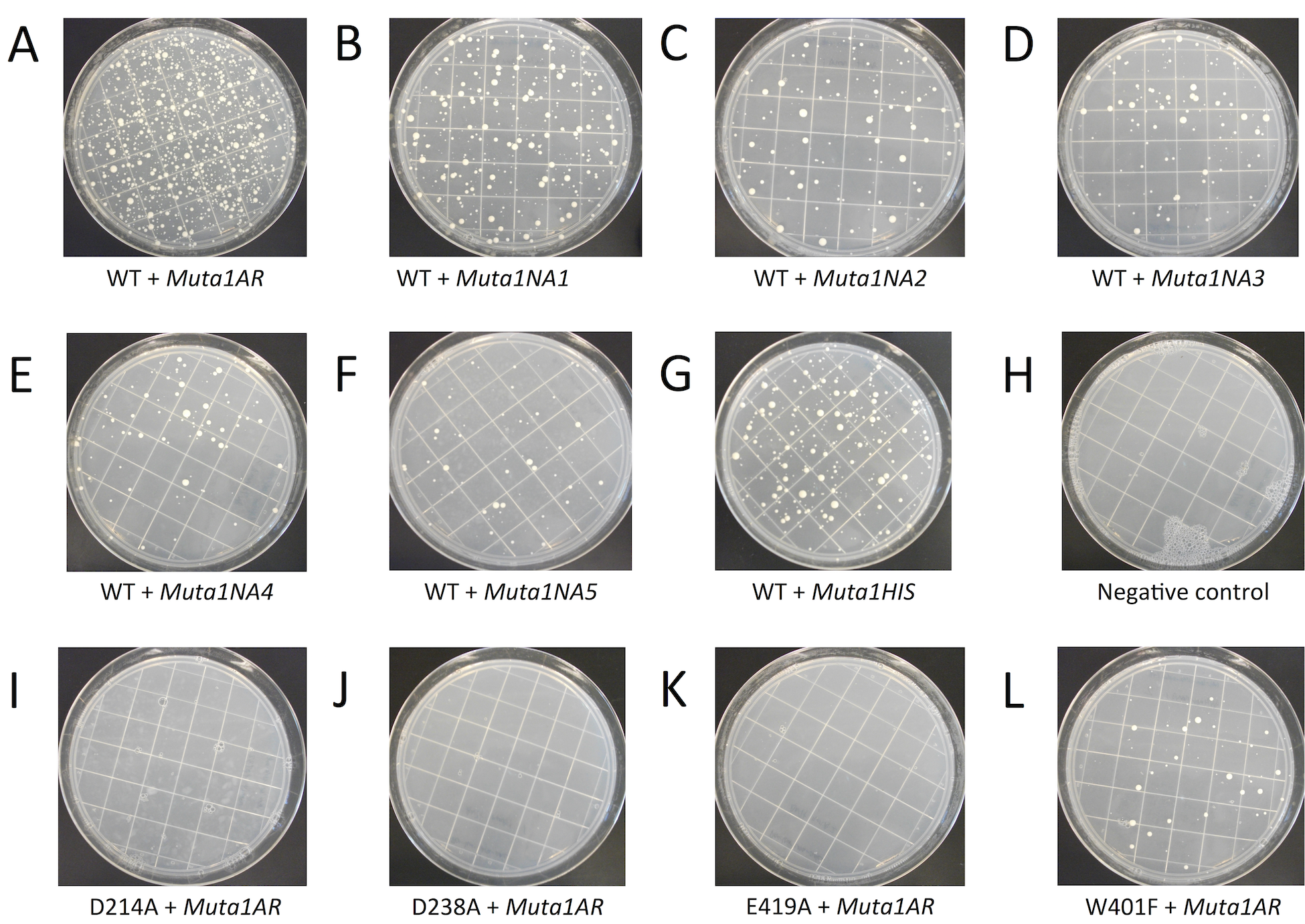

Supplement: Additional file 5: Figure S4. — ADE2 revertant colonies from the yeast transposition assay. (A-G) Excision activity of nonautonomous elements from the ADE2 coding region. (H) Negative control, Muta1AR excision from the ADE2 coding region is tested on plates without galactose. (I-L) Muta1AR excision from the ADE2 coding region is tested with mutant transposases. (TIF 3512 kb) [file 13100_2016_84_MOESM5_ESM.tif]

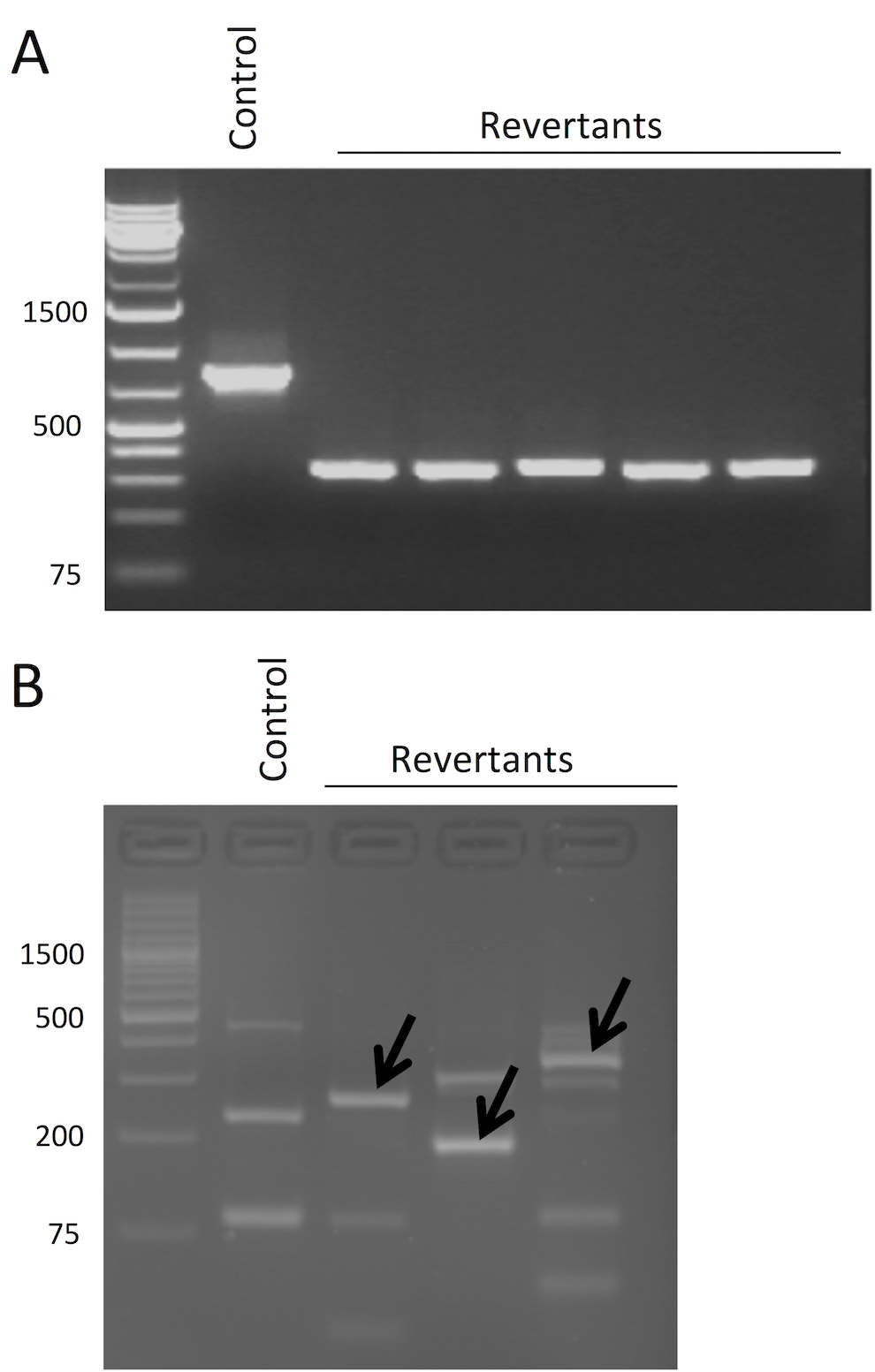

Supplement: Additional file 6: Figure S5. — Analysis of excision and reinsertion events. (A) PCR analysis of the Muta1NA1 excision sites from ADE2 revertants using flanking primers. Expected band size is 820bp (control) or 350bp, with or without Muta1NA1, respectively. (B) Transposon display analysis of Muta1HIS reinsertion in the yeast genome. DNA bands are amplicons consisting of flanking sequences of the reinsertion sites and part of the TIR. PWL89A-Muta1HIS vector is used as control. Arrows indicate the polymorphic bands that represent the insertion of Muta1HIS in different genomic locations. (TIF 413 kb) [file 13100_2016_84_MOESM6_ESM.tif]

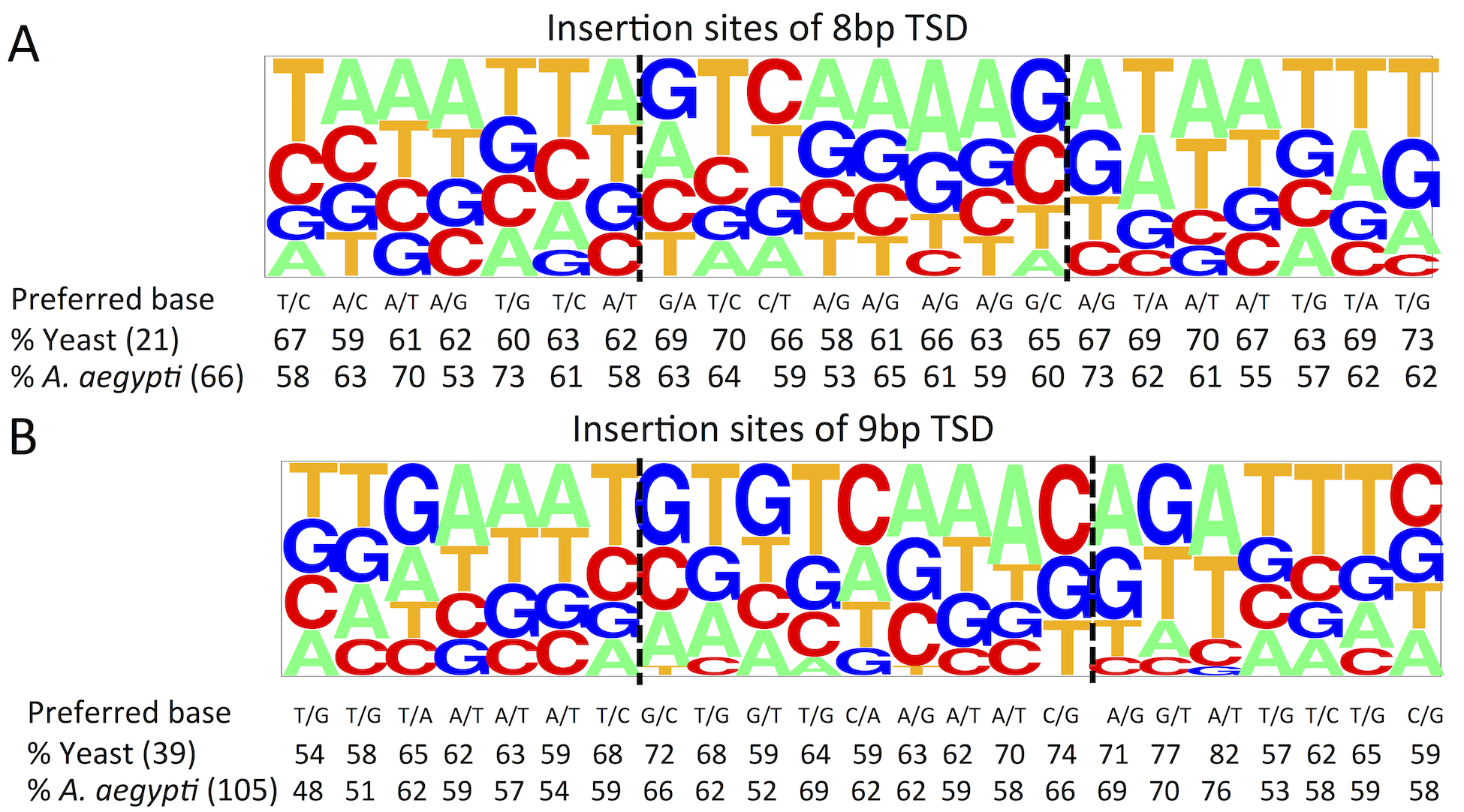

Supplement: Additional file 8: Figure S6. — Seqlogo of insertion sites of Ae. aegypti Muta1 derivative elements and reintegration sites in yeast. Both 8bp TSDs (A) and 9bp TSDs (B) and their 7bp flanking sequences are analyzed, insertion preference is shown as a pictogram (height of letter indicates percentage of each nucleotide at that position) and the frequencies of preferred nucleotides, if any, are shown. (TIF 1361 kb) [file 13100_2016_84_MOESM8_ESM.tif]

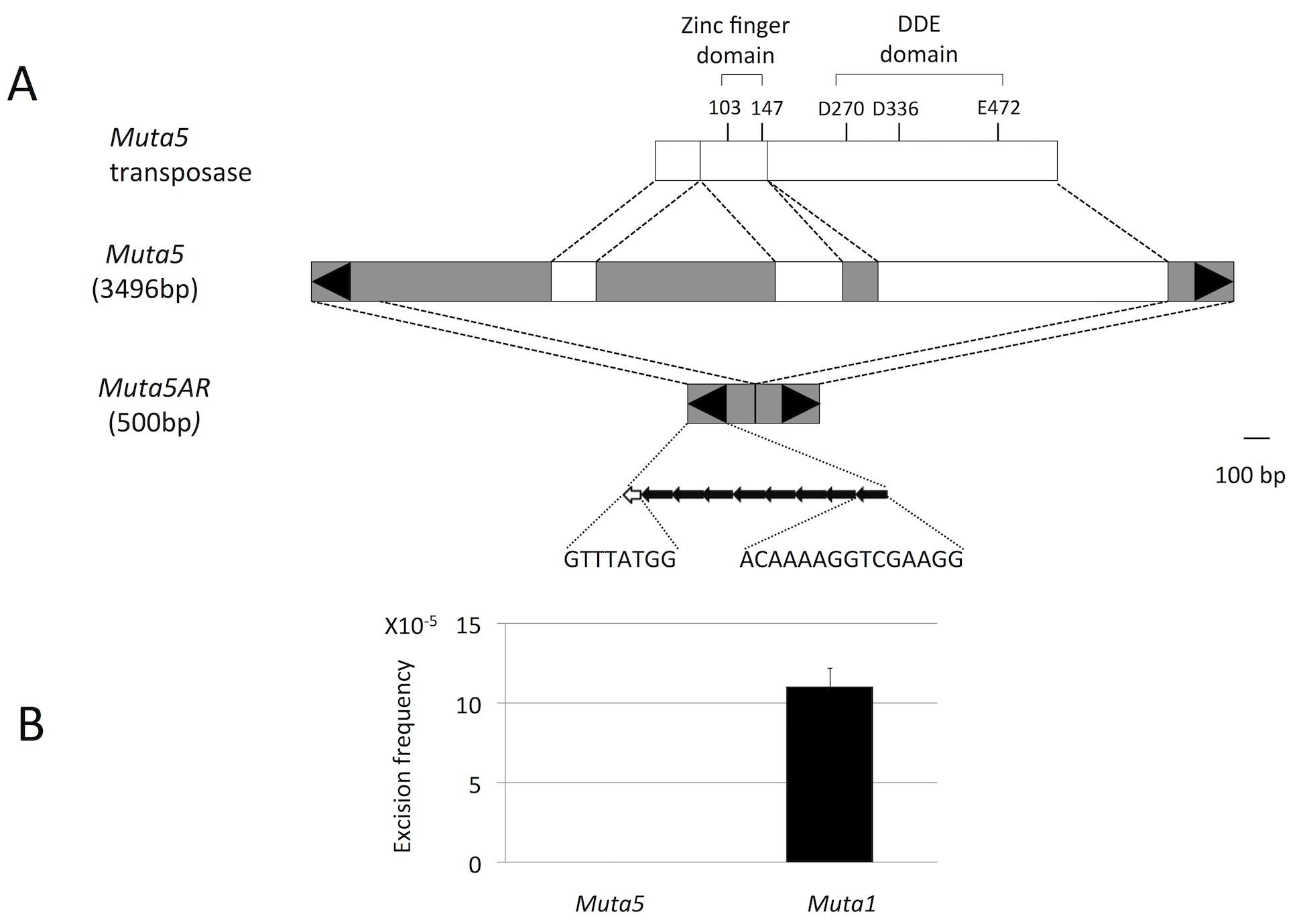

Supplement: Additional file 9: Figure S7 — Structural features of Muta5 and excision assay results. (A) Structural features of Muta5. White boxes are coding regions, shaded boxes are noncoding regions, and triangles are the TIR. Within the TIR, black arrows represent 9 copies of the 15bp subterminal tandem repeat, open arrow represents the 8bp terminal motif. The putative 554-residue transposase is predicted to harbor a zinc finger domain and the catalytic (DDE) domain. The artificial Muta5AR element contains 250 bp from each end of Muta5. (B) Excision frequencies of Muta5AR and Muta5AR from the ADE2 reporter in the yeast assay. Muta1 serves as the positive control. (TIF 373 kb) [file 13100_2016_84_MOESM9_ESM.tif]

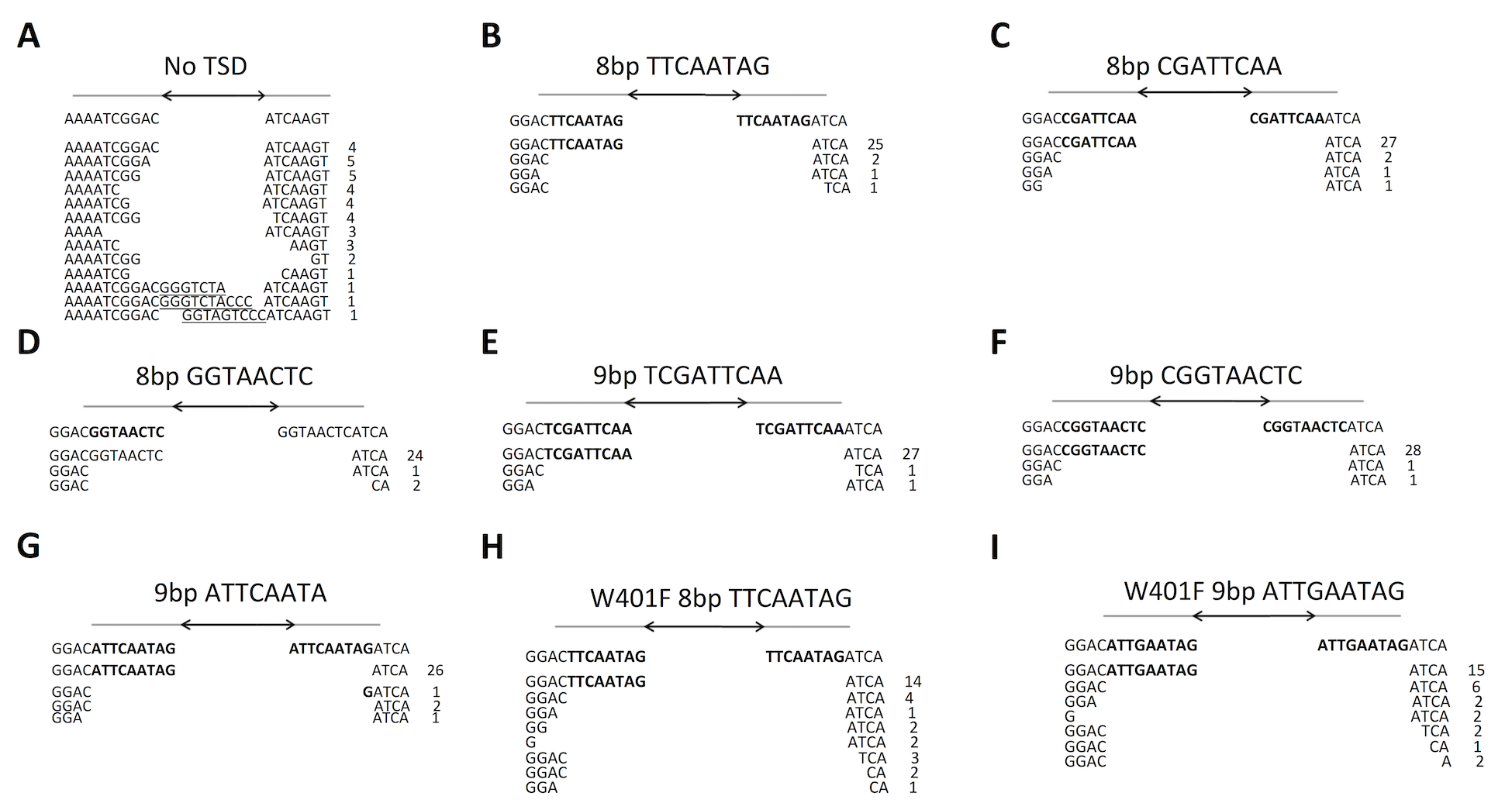

Supplement: Additional file 10: Figure S8. — Footprints from Muta1NA1 excision events. Arrows indicate the Muta1NA1 insertion, length and sequence of the TSD in each assay (shown above the arrows). TSD or sequences derived from TSDs are in bold, sequences derived from Muta1NA1 are underlined, the number of recovered events is on the right. (A) Footprints of Muta1NA1 excision from the ADE2 5’ UTR without donor site TSD. (B-D) Footprints of Muta1NA1 excision from the ADE2 5’ UTR with different 8 bp TSD sequences. (B) TTCAATAG; (C) CGATTCAA; (D) GGTAACTC. (E-G) Footprints of Muta1NA1 excision from the ADE2 5’ UTR with different 9 bp TSD sequences. (E) TCGATTCAA, (F) CGGTAACTC, (G) ATTCAATAG. (H-I) Footprints of Muta1NA1 excision from the ADE2 5’UTR with the transposase W401F mutation. (H) the 8bp TSD TTCAATAG was used. (I) the 9bp TSD ATTGAATAG was used. (TIF 665 kb) [file 13100_2016_84_MOESM10_ESM.tif]
