# Supplementary material for: Functional characterization of the active Mutator-like transposable element, Muta1 from the mosquito Aedes aegypti
Source: Mob DNA. 2017 Jan 11;8:1. doi: 10.1186/s13100-016-0084-6 (PMC5225508; doi:10.1186/s13100-016-0084-6)
Supplement: Additional file 2: Table S1. — Summary of 14 MULE families in Ae. aegypti. (DOCX 81 kb) [file 13100_2016_84_MOESM2_ESM.docx]

Table S1: Summary of 14 MULE families in *A. aegypti*.

| Family | Copy number | Full-length  copy | Length  (bp) * | Transposase  (Aa) ** | TIR length  (bp) *** | Subterminal repeats | No. of derivative elements |  |
| --- | --- | --- | --- | --- | --- | --- | --- | --- |
|  |  |  |  |  |  |  |  |  |
| Muta1 | 8 | 8 | 3198 | 504 | 145 | Present | 326 |  |
| Muta2 | 3 | 1 | 5258 | 531 | 174 | Present | 249 |  |
| Muta3 | 6 | 3 | 6738 | 488 | 16 | Absent | 103 |  |
| Muta4 | 2 | 1 | 6208 | 536 | 118 | Present | 88 |  |
| Muta5 | 7 | 4 | 3497 | 554 | 151 | Present | 210 |  |
| Muta6 | 3 | 1 | 2447 | 416 | 223 | Absent | 0 |  |
| Muta7 | 4 | 3 | 4167 | 463 | 228 | Absent | 77 |  |
| Muta8 | 2 | 1 | 2661 | 467 | 116 | Present | 54 |  |
| Muta9 | 3 | 2 | 3273 | 518 | 117 | Present | 298 |  |
| Muta10 | 3 | 2 | 6708 | 510 | 156 | Present | 266 |  |
| Muta11 | 2 | 1 | 3231 | 491 | 160 | Present | 48 |  |
| Muta12 | 4 | 1 | 2844 | 470 | 59 | Absent | 85 |  |
| Muta13 | 2 | 1 | 3671 | 510 | 131 | Present | 102 |  |
| Muta14 | 2 | 2 | 2768 | 534 | 115 | Present | 132 |  |

* For families with multiple full-length elements, length is based on element with the longest transposase.

** For families with multiple full-length elements, the longest transposase among family members is used here.

*** For families with multiple full-length elements, TIR length is based on element with the longest transposase.
