# Supplementary material for: Functional characterization of the active Mutator-like transposable element, Muta1 from the mosquito Aedes aegypti
Source: Mob DNA. 2017 Jan 11;8:1. doi: 10.1186/s13100-016-0084-6 (PMC5225508; doi:10.1186/s13100-016-0084-6)
Supplement: Additional file 7: Table S2. — Target Site Duplications (TSDs) and locations of Muta1 transpositions in yeast. (DOCX 96 kb) [file 13100_2016_84_MOESM7_ESM.docx]

**TableS2. Target Site Duplications (TSDs) and locations of *Muta1* transpositions in yeast.**

| Insertion site | Target TSD length (bp) | Target TSD sequence |
| --- | --- | --- |
| intergenic | 8 | CCTACCGA |
| intergenic | 8 | TTTCGTAG |
| intergenic | 8 | CCGGTTGA |
| intergenic | 8 | CTCTTGTT |
| intergenic | 8 | TTGATCAA |
| intergenic | 8 | GCATGGAA |
| intergenic | 8 | AGCTACAA |
| intergenic | 8 | AGACCAAT |
| intergenic | 8 | CTCTTTGC |
| intergenic | 9 | CAGGGCTGC |
| intergenic | 9 | TGCATGATA |
| intergenic | 9 | AAATTGATA |
| intergenic | 9 | CTCCCACAA |
| intergenic | 9 | TCCACCAAT |
| intergenic | 9 | GTGGGAATC |
| intergenic | 9 | TTCGCGGAG |
| intergenic | 9 | GTACTCCTT |
| intergenic | 9 | ATATCGTCA |
| intergenic | 9 | GGATAGTTT |
| within CDC15 gene | 8 | TGCGTCGT |
| within FLO9 gene | 8 | GTCCACCA |
| within PYC1 gene | 8 | ATTGGCTT |
| within PRP1 gene | 8 | TATTGTCC |
| within ERG13 gene | 8 | TTGGCTCT |
| within ATS1 gene | 9 | CCTATTATG |
| within JEN1 gene | 9 | CTGTACTCC |
| within YAP1801 gene | 9 | TTTCAATTG |
| within AIM39 gene | 9 | CAGAATGAG |
| within KIN2 gene | 9 | AGTGCGCTG |
| within TAX4 gene | 9 | AGCAAGAGT |
| within AFG2 gene | 9 | CTGGGTGTG |
| within GTT1 gene | 9 | TTCTTACCA |
| within MRPS35 gene | 9 | AACAGACGG |
| 5' to TAT1 gene | 8 | ATGCAGCA |
| 5' to CHZ1 gene | 8 | TTGATGGG |
| 5' to HAP4 gene | 8 | AGTCTAGC |
| 5' to RSC58 gene | 8 | CCATCTGG |
| 5' to THI3 gene | 9 | GGGGCTGAT |
| 5' to UPF3 gene | 9 | GAATAGTGA |
| 5' to OTU2 gene | 9 | GCATATCTC |
| 5' to SET5 gene | 9 | GCCATCTTC |
| 5' to SMF3 gene | 9 | CAGCTCCAA |
| 5' to SPT21 gene | 9 | CAAGGGCTC |
| 5' to WWM1 gene | 9 | CTGTGCTTG |
| 5' to CLB3 gene | 9 | CGGCTTAGA |
| 5' to HFM1 gene | 9 | AAAGTATTT |
| 5' to MUK1 gene | 9 | GAATGTTCT |
| 5' to VMA9 gene | 9 | GAGAGTGAC |
| 5' to AQY2 gene | 9 | TAAGCATTG |
| 3' to IRC20 gene | 8 | CATCCGAA |
| 3' to VRG4 gene | 8 | CTGTTTTC |
| 3' to STE12 gene | 8 | TTCCTGTA |
| 3' to KGD1 gene | 9 | CTAACCTCG |
| 3' to PCL6 gene | 9 | GCTCTCATT |
| 3' to SRS2 gene | 9 | GGCTAACAG |
| 3' to SAM50 gene | 9 | GCCTCACTA |
| 3' to IRC5 gene | 9 | TCCAGTATC |
| 3' to ABP1 gene | 9 | TCCCTTTCA |
| 3' to STE11 gene | 9 | TACCCCTGG |
| 3' to GPH1 gene | 9 | ATTGGTACA |
