# Supplementary material for: Functional characterization of the active Mutator-like transposable element, Muta1 from the mosquito Aedes aegypti
Source: Mob DNA. 2017 Jan 11;8:1. doi: 10.1186/s13100-016-0084-6 (PMC5225508; doi:10.1186/s13100-016-0084-6)
Supplement: Additional file 11: Table S3. — Primers used in this study. (DOCX 124 kb) [file 13100_2016_84_MOESM11_ESM.docx]

Table S3 Primers used in this study

| Primers for amplifying *Muta1* coding sequences | | |
| --- | --- | --- |
| Muta1_out_L |  | TCTGGAGGGTTGATTGTTTG |
| Muta1_out_R |  | CTGAAGGTGGTCCGTCTTAC |
| Muta1_exon1_L | | CACCATGGACTCGGACAGCGAT |
| Muta1_exon1_R | | CAGTAGTGGGTGGGACCATTCGGTGTTCTGTTTGAATAGA |
| Muta1_exon2_L | | TCTATTCAAACAGAACACCGAATGGTCCCACCCACTACTG |
| Muta1_exon2_R | | TTATTTTGATTTTGATCCTAAGTGA |
| Primers for amplifying nonautonomous elements | | |
| Muta1AR_LL |  | GGGTCTACCCCGTTTGGC |
| Muta1AR_LR |  | CCAATTCATTTATTATTTCAATTTTTTGACAAAGTGACCCATAACAG |
| Muta1AR_RL |  | CTGTTATGGGTCACTTTGTCAAAAAATTGAAATAATAAATGAATTGG |
| Muta1AR_RR |  | GGGTCTACCCCGTTTGGC |
| Muta1NA1_L |  | CCCCTTATTTCATTAGTGTAGC |
| Muta1NA1_R |  | ATCGCATAGATTTGGTTGGT |
| Muta1NA2_L |  | AGTGGACTCCTTGCGCATGC |
| Muta1NA2_R |  | CAGGTGGACTCATCCGTGCC |
| Muta1NA3_L |  | GAACTTCCTGTTCTATTGAACTTCC |
| Muta1NA3_R |  | ACCCTCAGTCTTGTTTACGGCG |
| Muta1NA4_L |  | GATAGATAGGTAGATAGGGTCTACCCCG |
| Muta1NA4_R |  | GGACCAGTGTGCATTGTCGC |
| Muta1NA5_L |  | ACAGCGCAGTTCGCTCCTTG |
| Muta1NA5_R |  | GGAGATATAACTTAGGAGGGCCG |
| Primers for constructing *Muta1HIS* | | |
| Muta1HIS_L |  | CACACTGTTATGGGTCACTTTGTCACTCTTGGCCTCCTCTAGTAC |
| Muta1HIS_R |  | ATTTTTTGACAAAGTGACCCATAACAGGTATCATACTGTTCGTATAC |
| Primers for inserting elements in *ADE2* gene | | |
| ADE2_exon_IN_L | | ATTTACTAAAGAATTAGCAGTCATGATTGTGAGGTCTGTTGGGTCTACCCCGTTTGGC |
| ADE2_exon_IN_R | | GGATAGTCTCTACAATTGGGTAAGAAAACACTAAACCGTTGGGTCTACCCCGTTTGGC |
| ADE2_UTR_IN_L | | ACTATAACAATCAAGAAAAACAAGAAAATCGGACCTCGAGGGGTCTACCCCGTTTGGC |
| ADE2_UTR_IN_R | | TAATATACCAACTGTTCTAGAATCCATACTTGATCTCGAGGGGTCTACCCCGTTTGGC |
| ADE2_UTR_8bpAL | | ACTATAACAATCAAGAAAAACAAGAAAATCGGACCTCGAGTTCAATAGGGGTCTACCCCGTTTGGC |
| ADE2_UTR_8bpAR | | TAATATACCAACTGTTCTAGAATCCATACTTGATCTCGAGCTATTGAAGGGTCTACCCCGTTTGGC |
| ADE2_UTR_8bpBL | | ACTATAACAATCAAGAAAAACAAGAAAATCGGACCTCGAGCGATTCAAGGGTCTACCCCGTTTGGC |
| ADE2_UTR_8bpBR | | TAATATACCAACTGTTCTAGAATCCATACTTGATCTCGAGTTGAATCGGGGTCTACCCCGTTTGGC |
| ADE2_UTR_8bpCL | | ACTATAACAATCAAGAAAAACAAGAAAATCGGACCTCGAGGGTAACTCGGGTCTACCCCGTTTGGC |
| ADE2_UTR_8bpCR | | TAATATACCAACTGTTCTAGAATCCATACTTGATCTCGAGGAGTTACCGGGTCTACCCCGTTTGGC |
| ADE2_UTR_9bpAL | | ACTATAACAATCAAGAAAAACAAGAAAATCGGACCTCGAGATTCAATAGGGGTCTACCCCGTTTGGC |
| ADE2_UTR_9bpAR | | TAATATACCAACTGTTCTAGAATCCATACTTGATCTCGAGCTATTGAATGGGTCTACCCCGTTTGGC |
| ADE2_UTR_9bpBL | | ACTATAACAATCAAGAAAAACAAGAAAATCGGACCTCGAGTCGATTCAAGGGTCTACCCCGTTTGGC |
| ADE2_UTR_9bpBR | | TAATATACCAACTGTTCTAGAATCCATACTTGATCTCGAGTTGAATCGAGGGTCTACCCCGTTTGGC |
| ADE2_UTR_9bpCL | | ACTATAACAATCAAGAAAAACAAGAAAATCGGACCTCGAGCGGTAACTCGGGTCTACCCCGTTTGGC |
| ADE2_UTR_9bpCR | | TAATATACCAACTGTTCTAGAATCCATACTTGATCTCGAGGAGTTACCGGGGTCTACCCCGTTTGGC |
| Primers for amplifying excision empty site | | |
| ADE2_exon_EX_L | | GGAACAAGCCAGTGAGACG |
| ADE2_exon_EX_R | | TTGAGAAGTGACGCAAGCA |
| ADE2_UTR_EX_L | | TGATTGATTATTACAGCTATG |
| ADE2_UTR_EX_R | | TGTAGGAACATCAACATGCTC |

Primers for TE display

| TD_out |  | ATGCCAAACGGGGTAGACCC |  |  |  |
| --- | --- | --- | --- | --- | --- |
| TD_in |  | TTTAAAAATACGATTTCTGG |  |  |  |
| Bfa1 |  | GACGATGAGTCCTGAGTA |  |  |  |

Primers for transposase mutagenesis

| E129AL |  | GCCGGAGCAGCATTTTCTAATG | |  |  |
| --- | --- | --- | --- | --- | --- |
| E129AR |  | CATTAGAAAATGCTGCTCCGGC | |  |  |
| E188AL |  | CTGATATTAAGGCAGGAAGCGAC | |  |  |
| E188AR |  | GTCGCTTCCTGCCTTAATATCAG | |  |  |
| D214AL |  | GGATCGTTGCCGGAACGTTC |  |  |  |
| D214AR |  | GAACGTTCCGGCAACGATCC |  |  |  |
| E239AL |  | CCAAACCACGCACATGCGTTC |  |  |  |
| E239AR |  | GAACGCATGTGCGTGGTTTGG |  |  |  |
| D283AL |  | CATTTTATCAGCTTTTGAAAAGGC | |  |  |
| D283AR |  | GCCTTTTCAAAAGCTGATAAAATG | |  |  |
| H307AL |  | CGTGCTTCTTTGCCCTGTCCCAG | |  |  |
| H307AR |  | CTGGGACAGGGCAAAGAAGCACG | |  |  |
| W313AL |  | CCAGAATTTCGCGAAAAGAATT | |  |  |
| W313AR |  | CCTCTTGAATTCTTTTCGCGAA |  |  |  |
| W313FL |  | CCCAGAATTTCTTTAAAAGAATTCAA | |  |  |
| W313FR |  | GCCTCTTGAATTCTTTTAAAGAAA | |  |  |
| E347AL |  | TTTACCAACTGCACGTATACCAGC | |  |  |
| E347AR |  | GCTGGTATACGTGCAGTTGGTAAA | |  |  |
| W401AL |  | CCCGCCGTCGTTGGCGTCGATTTATG | |  |  |
| W401AR |  | CGTTGTCATAAATCGACGCCAACGAC | |  |  |
| W401FL |  | GCCGTCGTTGTTTTCGATTTATG | |  |  |
| W401FR |  | GTCATAAATCGAAAACAACGACGG | |  |  |
| E419AL |  | CAAACCAGATTGCAGCCTGGC |  |  |  |
| E419AR |  | GCCAGGCTGCAATCTGGTTTG |  |  |  |
| D473AL |  | CATCAAATTAATGCTCAAGCGGTG | |  |  |
| D473AR |  | CACCGCTTGAGCATTAATTTGATG | |  |  |
